# Epidemiological Factors Associated with Dengue Shock Syndrome and Mortality in Hospitalized Dengue Patients in Ho Chi Minh City, Vietnam

**DOI:** 10.4269/ajtmh.2011.10-0476

**Published:** 2011-01-05

**Authors:** Katherine L. Anders, Nguyen Minh Nguyet, Nguyen Van Vinh Chau, Nguyen Thanh Hung, Tran Thi Thuy, Le Bich Lien, Jeremy Farrar, Bridget Wills, Tran Tinh Hien, Cameron P. Simmons

**Affiliations:** Oxford University Clinical Research Unit, Ho Chi Minh City, Vietnam; Hospital for Tropical Diseases, Ho Chi Minh City, Vietnam; Department of Dengue Haemorrhagic Fever, Children's Hospital No. 1, Ho Chi Minh City, Vietnam; Department of Infectious Diseases, Children's Hospital No. 2, Ho Chi Minh City, Vietnam

## Abstract

Understanding trends in dengue disease burden and risk factors for severe disease can inform health service allocation, clinical management, and planning for vaccines and therapeutics. Dengue admissions at three tertiary hospitals in Ho Chi Minh City, Vietnam, increased between 1996 and 2009, peaking at 22,860 in 2008. Children aged 6–10 years had highest risk of dengue shock syndrome (DSS); however, mortality was highest in younger children and decreased with increasing age (odds ratio [OR] = 0.52, 95% confidence interval [CI] = 0.36–0.75 in 6- to 10- year-old children and OR = 0.27, 95% CI = 0.16–0.44 in 11- to 15-year-old children compared with 1- to 5-year-old children). Males were overrepresented among dengue cases; however, girls had higher risk of DSS (OR = 1.19, 95% CI = 1.14–1.24) and death (OR = 1.57, 95% CI = 1.14–2.17). Young children with dengue had greatest risk of death and should be targeted in dengue vaccine and drug trials. The increased risk of severe outcomes in girls warrants further attention in studies of pathogenesis, health-seeking behavior, and clinical care.

## Introduction

Dengue is a mosquito-borne acute systemic viral infection caused by any one of four serotypes of dengue virus (DENV). The ongoing dengue pandemic represents a major challenge to health systems in developing countries. Recent estimates of the disease burden suggest transmission occurs in up to 124 countries, with at least 35 million symptomatic cases per year and around 20,000 deaths.^1^ There are no licensed vaccines or anti-viral therapies for prevention or treatment, although these are in development.^2^,^3^

In Vietnam, dengue hemorrhagic fever was first identified in 1963 in the Mekong Delta region of southern Vietnam.^4^ Between 1963 and 1995, Vietnam reported 1,518,808 dengue hemorrhagic fever cases and 14,133 deaths.^5^ Data from the dengue surveillance program in southern Vietnam show epidemic peaks of increasing magnitude occurring approximately every 5 years between 1975 and 1987, with a longer gap of 11 years preceding a large epidemic of 119,429 dengue hemorrhagic fever (DHF) cases and 342 fatalities in 1998.^6^ It is not known whether age-related trends in dengue incidence in Vietnam are following a similar pattern to Thailand, where a striking increase in the average age of dengue cases has been observed.^7^ Furthermore, risk factors for severe and fatal dengue among hospitalized patients have not previously been described for Vietnam nor have they been documented extensively elsewhere. A better understanding of populations at high risk of complications and poor outcomes in endemic settings can inform the future implementation of dengue vaccines and anti-viral drugs.

The Hospital for Tropical Diseases (HTD) is the tertiary center for infectious diseases in Ho Chi Minh City (HCMC). Children's Hospitals Number 1 and Number 2 (CH1 and CH2) are the two main pediatric hospitals in HCMC. The aims of the current study were to characterize trends in the clinical and epidemiological characteristics of dengue cases admitted to these hospitals, to identify epidemiological risk factors for severe disease and death, and to discuss the implications of these observations for future clinical management, vaccines, and therapeutics.

## Materials and Methods

### Dengue patient and population data.

Data were obtained from the central hospital records of the HTD, CH1, and CH2 in Ho Chi Minh City, Vietnam, on the number of clinically diagnosed dengue cases admitted between 1996 and 2009. Anonymous addresses at the district level were also obtained from hospital records of dengue patients admitted in 2008 and 2009. Population data were obtained from the Ho Chi Minh City statistical office website.^8^

### Case definitions.

Dengue cases were diagnosed based on clinical signs, symptoms, and hematological tests and classified by severity grade according to the Vietnam Ministry of Health guidelines, which are adapted from the 1997 World Health Organization (WHO) guidelines.^9^ A discharge diagnosis was recorded for each patient according to the International Classification of Diseases, revision 10 (ICD-10). Only patients with a clinical diagnosis of dengue were included in the dataset for this study, but the coding applied to these patients varied slightly between the study hospitals. Dengue patients at HTD included those with an ICD-10 code for dengue fever (DF) or DHF at discharge. All clinically diagnosed dengue cases at CH1 received an ICD-10 code for DHF, whereas at CH2, all patients with a clinical diagnosis of dengue received an ICD-10 code for viral hemorrhagic fever. Modified ICD codes were recorded at discharge by the hospitals to distinguish between DHF severity grades, with DHF grades 3 and 4 classified as dengue shock syndrome (DSS).

### Diagnostic serology.

Since 2004, the HTD has used two formats of an immunoglobulin M (IgM) antigen capture ELISA for the presumptive laboratory diagnosis of dengue in a proportion of clinically suspected cases. During 2004 and 2005, cases were tested using a commercially available DENV IgM antigen-capture (MAC) enzyme-linked immunosorbent assay (ELISA; Inverness Medical, Brisbane, Australia). From 2006, an in-house MAC ELISA using reagents from Venture Technologies (Sarawak, Malaysia) has been used. The results of the diagnostic serology, where performed, were not linked to the central patient records; therefore, the laboratory data were analyzed separately from the main patient dataset.

### DENV serotype prevalence.

Since 1999, acute blood specimens have been collected from a subset of adult and pediatric dengue patients admitted to HTD and enrolled in various prospective clinical studies. These specimens were analyzed by reverse transcription polymerase chain reaction (RT-PCR), as described previously,^10^ for the determination of the infecting DENV serotype. These data have been published elsewhere^11^ and are reproduced as a supporting file to this manuscript.

### Dengue incidence data.

The Pasteur Institute of Ho Chi Minh City collates surveillance data on the number of dengue cases admitted to hospitals in the southern 20 provinces of Vietnam and produces annual estimates of the incidence of dengue per 100,000 population in southern Vietnam. These data have been published elsewhere^11^ and are reproduced with the permission of the Pasteur Institute HCMC for the purpose of comparison with the results of the current study.

### Data analysis.

All analyses were performed in SPSS for Windows version 14 (IBM, Somers, NY) and Stata/IC version 11 (Stata Corp., College Station, TX). χ^2^ test statistics were calculated for comparisons of proportions between groups. A non-parametric test of the equality of medians was used for comparisons of non-normally distributed continuous variables between groups. The relationship between two continuous variables was analyzed by linear regression. Logistic regression was used to model the association between patient characteristics and the occurrence of DSS or mortality. Variables that were significant at the < 0.05 level in univariate analysis were included in a multivariate logistic regression model. Two-sided *P* values < 0.01 were considered statistically significant in the multivariate model. Missing values were imputed in the multivariate model using Stata's mi impute command with the logistic regression method for binary variables. MapInfo geographical information software version 9 (Pitney Bowes Software Inc., Troy, NY) was used for geographic mapping of dengue cases.

## Results

### Case burden of dengue at three referral hospitals in HCMC.

**Figure 1.**
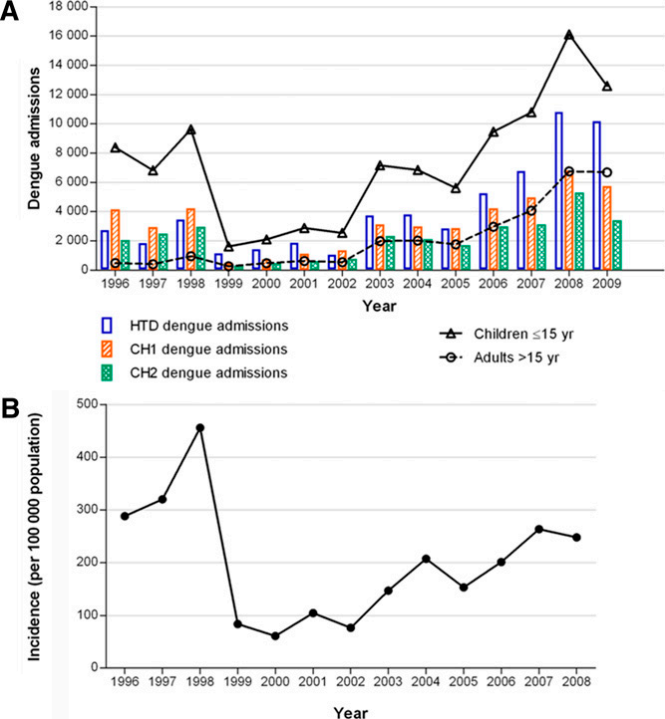
Temporal trends in dengue admissions in Ho Chi Minh City, Vietnam, from 1996 to 2008. (**A**) Bars show the number of in-patients with clinically diagnosed dengue each year at each of the three study hospitals. Lines show the number of dengue cases in children aged 15 years or less (triangles) and adults over the age of 15 (circles) combined across the study sites. (**B**) Shown for comparison is the annual incidence of hospitalized dengue per 100,000 people in the southern 20 provinces of Vietnam derived from cases reported through the southern Vietnam dengue surveillance system. Data collated by and reproduced with permission from the Pasteur Institute HCMC. This figure appears in color at www.ajtmh.org.

Between 1996 and 2009, dengue was clinically diagnosed in 132,480 patients admitted to one of the three study hospitals (56,965 at HTD, 45,275 at CH1, and 30,240 at CH2). The annual caseload of dengue at the three hospitals peaked at 10,579 in 1998 and again, in 2008, at 22,860 admissions after increasing steadily over the previous decade (Figure 1A). The trend in case load at the three study hospitals closely reflected the incidence of hospitalized dengue in southern Vietnam as reported by the Ministry of Health dengue surveillance program (Figure 1B).

A DENV-IgM laboratory test was performed in 29.7% (annual range = 11.1–41.0%) of clinically diagnosed dengue cases at HTD between 2004 and 2009. Of those tested, 71.7% (annual range = 63.8–75.5%) were serologically positive for DENV-IgM in a single specimen (Table 1). The proportion of tested patients with a positive IgM test result was higher if the specimen was collected ≥ 5 days after onset of illness (Table 1).

The relative prevalence of the four dengue virus serotypes among dengue patients admitted to HTD fluctuated over time, with periodic replacement of the dominant serotype approximately every 3 years (Supplemental Figure 1). DENV-1 has accounted for the vast majority of infections during the exceptionally high case burden years between 2007 and 2009.

### Trends in the age distribution of dengue patients.

**Figure 2.**
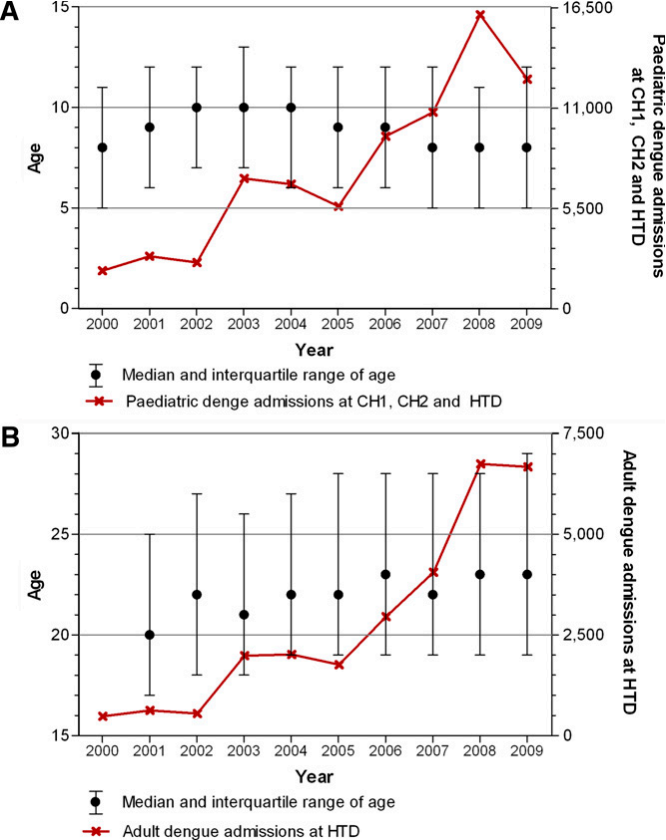
Age distribution of hospitalized dengue cases. (**A**) Circles show the median and lines show the interquartile range of the age of pediatric dengue patients admitted to Children's Hospitals 1 and 2 (CH1 and CH2) and the Hospital for Tropical Disease (HTD) each year from 2000 to 2009. In 2000, there was no data from HTD. The solid line indicates the total number of pediatric admissions for dengue (≤ 15 years) across the three hospitals each year. (**B**) Circles show the median and lines show the interquartile range of the age of adult dengue patients admitted to HTD each year from 2001 to 2009. The solid line indicates the total number of adult admissions for dengue (> 15 years) at HTD each year. This figure appears in color at www.ajtmh.org.

All dengue patients at CH1 and CH2 were children aged ≤ 15 years. Adults represented an increasing proportion of the dengue patient population at HTD over time (Figure 1A), from 18% in 1996 to 66% in 2009. Data on the precise age of dengue cases were available since 2000 at CH1 and CH2 and since 2001 at HTD. The median age of pediatric dengue patients admitted to the three study hospitals declined from 10 years of age in 2002–2004 to 8 years of age in 2007–2009 (Figure 2A) (estimated change in median age of pediatric patients per year from 2002 to 2009 = −0.36 years, 95% confidence interval [CI] = −0.48 to −0.23 years). The age distribution of pediatric cases in 2000 was similar to that seen in 2007–2009. In contrast, the median age of adult dengue patients admitted to HTD has increased steadily over the past decade, from 20 years in 2001 to 23 years in 2009 (Figure 2B) (estimated change in median age of adult patients per year from 2001 to 2009 = 0.30 years, 95% CI = 0.11–0.49 years).

### Gender bias in the dengue case burden.

**Figure 3.**
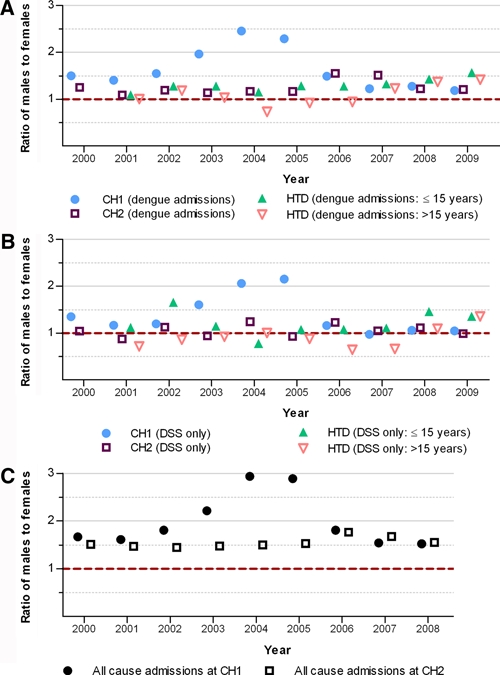
Male to female ratio of dengue patients and total hospital admissions. Points indicate the male to female ratio each year for (**A**) total dengue admissions, (**B**) cases of dengue shock syndrome, and (**C**) all-cause admissions by hospital: Children's Hospital Number 1 (CH1; circles), Children's Hospital Number 2 (CH2; squares), and Hospital for Tropical Diseases (HTD; triangles). The dashed line indicates an equal ratio of males and females. This figure appears in color at www.ajtmh.org.

Fifty-seven percent of the pediatric dengue patients admitted to the three study hospitals between 1996 and 2009 were male, and this gender bias was observed each year at each of the hospitals (Figure 3A). Among adult dengue patients admitted to HTD during the study period, 54% were male. Among pediatric patients with DSS, the gender bias was somewhat reduced but still apparent (55% overall; annual range = 50–61%) (Figure 3B).

To determine whether this predominance of males was specific to dengue patients, we obtained data on the sex distribution of all admissions to CH1 and CH2 from 2000 to 2008. The gender bias to males was even more pronounced for total admissions than for dengue (Figure 3C). Of almost 1 million admissions at the two pediatric hospitals between 2000 and 2008, 63% (594,293 of 941,888) were male. This bias was observed consistently each year and at both hospitals, with a minimum proportion of males of 59% at CH2 in 2002 and a maximum of 75% at CH1 in 2004 (Figure 3C). The bias to males was particularly marked in 2003–2006.

### DSS and case fatality rate.

**Figure 4.**
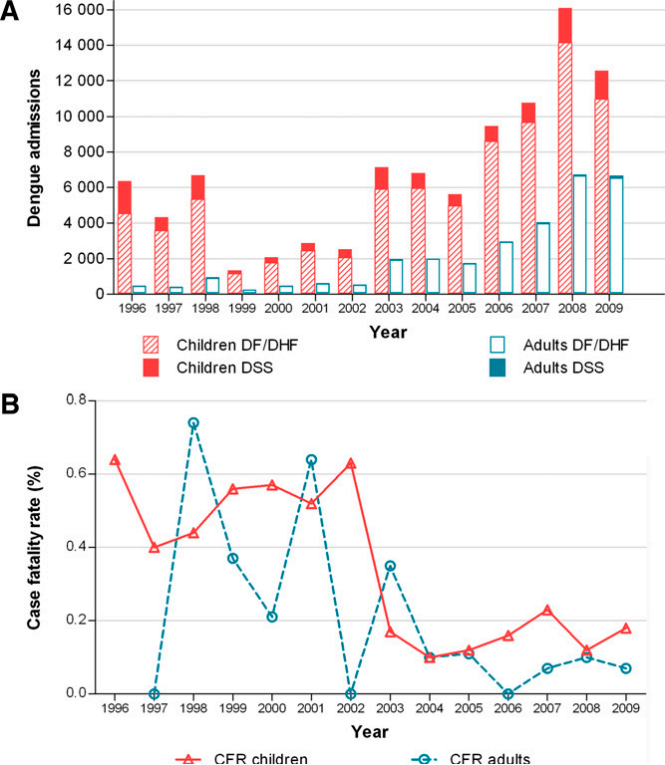
Disease severity and mortality among dengue patients. (**A**) Bars show the number of dengue patients admitted to the three study hospitals each year from 1996 to 2009 by disease severity. Red bars show pediatric DF/DHF (striped) and DSS (solid) cases. White and blue bars show adult DF/DHF (white) and DSS (blue) cases. Note that 1996 to 1999 excludes data from Children's Hospital Number 2, because figures on disease severity were not available. (**B**) Lines show the case fatality rate among all pediatric (≤ 15 years; solid line) and adult (> 15 years; dotted line) dengue patients admitted to the three study hospitals each year from 1996 to 2009. This figure appears in color at www.ajtmh.org.

DSS is a pathognomonic and severe life-threatening complication of dengue. A total of 14,079 DSS patients were diagnosed at HTD, CH1, and CH2 between 1996 and 2009. These figures exclude any DSS cases at CH2 between 1996 and 1999, because data on disease severity were not available. The vast majority (96.6%) of all DSS cases between 1996 and 2009 were children. Overall, 14.3% of children with a clinical diagnosis of dengue at the three hospitals had DSS (annual range = 9.2–28.5%) compared with 1.6% (annual range = 0.6–6.7%) of adult dengue cases. No trend was observed in the proportion of dengue cases manifesting as DSS over time (Figure 4A).

The overall case fatality rate (CFR) within the study population between 1996 and 2009 was 0.25% (325 of 132,480). Annual case fatality rates ranged from 0.10% in 2004 to 0.64% in 1996, with a clear decreasing trend over time (Figure 4B) (average change in CFR per year = −0.04%, 95% CI = −0.06% to −0.02%). As expected, the majority of deaths occurred in patients with DSS: the CFR among DSS patients was 1.6% (153/9,784) compared with 0.03% (28/92,683) among clinically diagnosed dengue patients that did not have DSS.

Overall, mortality was higher in children than in adult dengue patients (CFR 0.20% versus 0.11%; *P* = 0.002), but among those adults with DSS (*N* = 484), mortality was much higher than in pediatric DSS patients (CFR 5.5% versus 1.4%; *P* < 0.001).

### Demographic risk factors for DSS.

A better understanding of risk factors for DSS is important for understanding its pathogenesis and for the introduction of new prevention strategies, such as vaccines. To this end, we analyzed the available demographic characteristics of the 75,057 pediatric dengue patients admitted to CH1 and CH2 between 2000 and 2009 and to HTD between 2001 and 2009. Sex, age group, hospital, location of residence (HCMC or province), year, and season of admission (dry or wet) were all associated with DSS by univariate analysis (*P* < 0.001). In a multivariate logistic regression model (Table 2), with the exception of season of admission, each of these variables was independently associated with DSS after adjusting for the model covariates. The risk of DSS had a non-linear relationship with age, with shock more prevalent among pediatric dengue patients aged 6–10 years than younger age groups or older children. A greater proportion of girls than boys had DSS; however, males still represented the majority of pediatric DSS patients because of their overall predominance in case numbers (Table 2).

### Risk factors for mortality.

Univariate and multivariate analyses of variables associated with mortality were performed first with a denominator of dengue patients of all severities (Table 3) and then within DSS patients only (Table 4). The multivariate models were fitted with the same covariates as the DSS model: sex, age group, hospital, location of residence, year, and season of admission. With the exception of season of admission, all of these variables were significantly associated with mortality by univariate analysis (*P* < 0.001). Although girls were underrepresented among DSS patients, they had a significantly higher mortality than boys, both as a proportion of all dengue cases (adjusted odds ratio [AOR] = 1.57, 95% CI = 1.14–2.17) and among DSS cases (AOR = 1.51, 95% CI = 1.06–2.15). Although children over the age of 5 years were more likely to have DSS, younger children had poorer outcomes. Both total mortality and mortality associated with DSS peaked in children aged 1–5 years and declined with age (6–10 years AOR = 0.40, 95% CI = 0.27–0.59 and 11–15 years AOR = 0.28, 95% CI = 0.16–0.48 compared with 1–5 years AOR and CI). Indeed, it is striking that the risk of death is approximately four times higher in a child aged 1–5 years than a child aged 11–15 years. The odds of mortality were two times as high among DSS patients resident outside Ho Chi Minh City compared with city residents (AOR = 2.10, 95% CI = 1.43–3.10) and among DSS patients at CH1 compared with HTD (AOR = 1.94, 95% CI = 1.04–3.61) or CH2.

### Minimum incidence of dengue in HCMC in 2008–2009.

**Figure 5.**
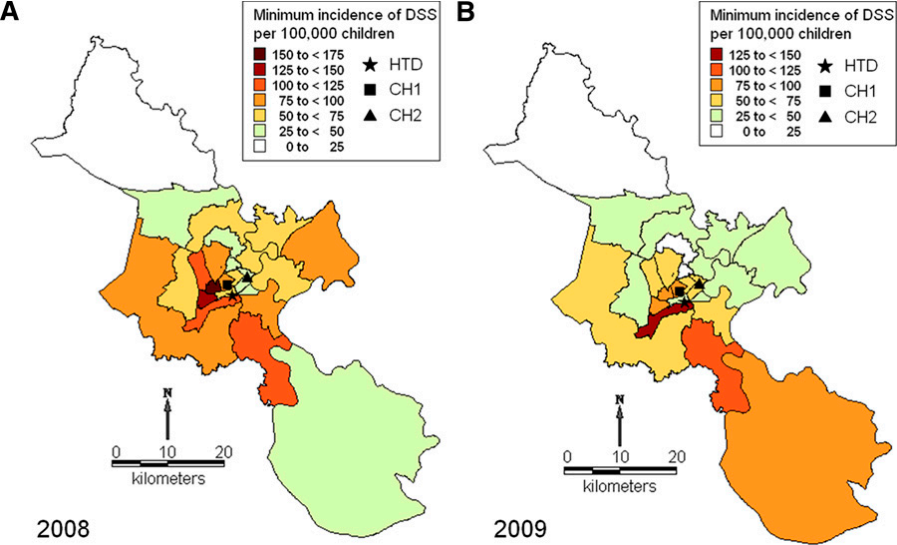
District-level minimum incidence of dengue shock syndrome in HCMC. Maps showing the estimated minimum incidence of DSS per 100,000 children in each district of HCMC in (**A**) 2008 and (**B**) 2009 based on DSS cases hospitalized at one of the three study hospitals and district-level population data. The locations of the three study hospitals are also shown: Hospital for Tropical Diseases (HTD ★), Children's Hospital Number 1 (CH1 ▪), and Children's Hospital Number 2 (CH2 ▴). This figure appears in color at www.ajtmh.org.

Using the dengue case burden documented at the three hospitals and district-level population data for HCMC as a denominator, we calculated a minimum estimate of the annual incidence of dengue requiring hospitalization in 2008 and 2009. In HCMC as a whole, 251 per 100,000 people in 2008 and 199 per 100,000 people in 2009 were admitted to one of the three study hospitals with a clinical diagnosis of dengue. We also estimated the minimum incidence in children aged 15 years and under. In 2008, 712 children per 100,000 in HCMC were hospitalized with suspected dengue, and remarkably, in four districts, this exceeded 1,000 per 100,000 or 1% of children. In 2009, at least 518 per 100,000 children in HCMC were hospitalized with suspected dengue. Because this estimate includes a proportion of patients who may not have had a true dengue virus infection, we also calculated a minimum estimate of the annual incidence of DSS in children in HCMC by district of residence, because the clinical diagnosis for DSS has much higher specificity than for non-shock dengue. In 2008, 74 per 100,000 children resident in HCMC were admitted to one of the three study hospitals with DSS, and the incidence of DSS in individual HCMC districts ranged from 7 to 152 per 100,000 children (Figure 5A). In 2009, the minimum estimate for DSS incidence was 57 per 100,000 children for HCMC as a whole and ranged from 15 to 127 per 100,000 children in individual districts of HCMC (Figure 5B).

## Discussion

This retrospective study of more than 100,000 patients describes the epidemiological profile of dengue cases admitted to three large hospitals in Ho Chi Minh City, Vietnam, between 1996 and 2009. Against a backdrop of surging incidence and oscillations in virus serotype, we show that girls and very young children hospitalized with a clinical diagnosis of dengue are at the highest risk of a poor outcome, and this has implications for implementation of vaccines and therapeutics.

A steep increase in the adult and pediatric dengue case burden is apparent at each of the study hospitals in HCMC over the last decade. The majority of the combined dengue burden at the three hospitals remains in children under the age of 15 years; however, adults now represent an increasing majority of the caseload at HTD. The median age of adult cases at HTD increased steadily from 20 to 23 years between 2001 and 2009. Although this observation is from one hospital and may not be a general trend, to our knowledge, there has not been any change in referral policy or care-seeking behavior that alone explains such a substantial increase in the adult dengue patient population at this hospital. It is possible that this reflects migration in adulthood from non- or less-endemic areas of Vietnam to this high transmission setting, where adults are disproportionally more likely to be symptomatic on infection.^7^,^12^ In Thailand, a substantial increase in the average age of dengue cases has occurred over the past 20 years, and this has been attributed to demographic changes, leading to an aging population and a reduced force of infection.^7^ However, the absence of any sustained increase in the median age of Vietnamese pediatric cases and the increase in population-based incidence estimated from surveillance data suggest that the falling dengue transmission intensity reported in Thailand is not currently replicated in southern Vietnam.

Our dataset represents patients admitted to hospitals with a clinical diagnosis of dengue recorded at discharge. Clinically diagnosed dengue is not routinely laboratory confirmed in Vietnam or in many other endemic countries, and a clinical dengue diagnosis is the basis for routine reporting to national surveillance systems here as elsewhere. We accept that a proportion of the cases included in our analysis will not be true dengue cases, and we attempt to quantify this proportion using laboratory data from HTD, which shows that more than 70% of the clinically diagnosed dengue cases tested have detectable DENV-specific IgM indicative of an acute or very recent infection. Therefore, it is plausible that some of the patient characteristics that we identified as being associated with a diagnosis of DSS or overall mortality could be confounded by the imperfect accuracy of the clinical diagnosis. In contrast, the accuracy of a clinical diagnosis of DSS is extremely high: among 2,628 clinically diagnosed DSS patients enrolled in ongoing research studies at HTD (1999–2009), CH1, and CH2 (2008–2009), 98% were subsequently laboratory confirmed as dengue cases (Wills B and Simmons CP, unpublished data). Therefore, our results derived from the DSS patient population (*N* = 9,392 cases) are highly unlikely to be confounded by misdiagnosed cases.

DSS is primarily a complication of dengue in children, and we found no evidence to suggest that the increase in case prevalence among adults at HTD was associated with a parallel increase in the prevalence of DSS among this age group. When DSS did manifest in adults, it was associated with a higher mortality rate than in children with DSS. A review of fatal adult dengue cases at HTD found that almost all of these patients developed DSS before admission to HTD, usually on or after day 6 of illness, and often had been referred from a district or provincial hospital (Chau NVV, personal communication). Thus, higher mortality rates in adults with DSS likely reflect patient referral patterns and not necessarily true CFRs. Mortality within the study population was low and decreased substantially throughout the 14-year study period. The vast majority of fatal cases occurred in patients with shock, although the CFR within shock patients also decreased over time. We found that the age and sex of pediatric dengue patients were strongly associated with the risk of both DSS and mortality. Although both the prevalence and frequency of DSS was highest in children aged 6–10 years, younger children aged 1–5 years were at significantly higher risk of mortality than older children. This is consistent with population-based reports of age-specific morbidity and mortality in secondary DENV-2 infections (after DENV-1) occurring in an outbreak situation.^13^ However, to our knowledge, this is the first report to provide precise estimates of the age-specific relative risk of mortality among a large hospitalized pediatric dengue patient population. Knowledge of the age-specific risk of dengue is critical for rational implementation of dengue vaccines and anti-viral drugs. The substantial burden of severe disease and the increased risk of mortality in the youngest children support the delivery of dengue vaccines and potentially in the future, dengue anti-viral drugs to pre-school–aged children to achieve greatest impact on disease burden and mortality.

A substantial and unexpected bias to males was observed among dengue cases. This could not be explained by population demographics, because in urban Vietnam, the average male:female ratio in children is around 108:100 and in adults, is skewed to females.^14^ A biological basis for increased innate susceptibility to dengue in males is possible, but this has not been documented and is not evidenced in a number of smaller hospital-based studies that have variously reported no difference, a male excess, or a female excess in dengue patient populations.^15^–^19^ Differential exposure between males and females is a possible but unlikely cause of this gender distribution, especially in children. Healthcare-seeking behavior might account for the gender bias observed not only for dengue but also for total admissions at the pediatric hospitals, which saw more than two times as many boys as girls admitted in some years. The bias to males was particularly marked in 2003–2006; however, we are not aware of any changes in hospital policies or public health activities during this time that might explain a gender-specific change in healthcare-seeking behavior. Others have reported a gender disparity of similar magnitude among pediatric hospital admissions for all causes in Hong Kong,^20^ but the relative contribution of innate susceptibility and healthcare-seeking behavior remains unclear. Qualitative studies are warranted to further investigate factors influencing care-seeking behavior and triage decisions and the effect of these behaviors on access to healthcare and health outcomes for girls and women.

Interestingly, although girls were underrepresented among dengue patients of all severities, they experienced higher mortality than boys. A preponderance of females among severe dengue cases but not mild cases has been reported previously in two small studies^21^,^22^ and was suggested to reflect either a more robust cellular immune response or a higher intrinsic susceptibility to capillary permeability in females than males.^5^ The fact that a substantially higher mortality rate in females persists even within DSS cases alone suggests that there is a biological component to this gender-associated risk, although this could still be influenced by behavioral factors such as time of presentation or differences in the type of care provided to girls and boys.

Residence outside HCMC and admission to CH1 (compared with either HTD or CH2) were independently associated with a significantly higher prevalence of both DSS and mortality among our pediatric study population. This association probably reflects, in part, the different case mix of the hospitals and the referral patterns within HCMC and from the provinces. DSS patients that reside outside of HCMC may well have been referred late in their illness and with more severe disease. Although the data were not available to investigate this possibility, it highlights the pressing need for early diagnostics and prognostic markers of progression to severe disease so that appropriate monitoring, supportive treatment, and referral, if necessary, can be delivered in the earlier stage of illness.

In 2008, at least 1 in every 140 children in Ho Chi Minh City was admitted to one of the three study hospitals with a clinical diagnosis of dengue. This startlingly high disease burden underscores the failure of existing vector control strategies and the urgent need for vaccines. We observed a wide range in the district-level estimates of DSS incidence in children. Given the high specificity of a clinical diagnosis of DSS and the fact that the vast majority of DSS cases in HCMC are likely to be admitted to one of the three tertiary hospitals included in this study, either directly or by referral, this represents a reasonable estimate of the population incidence and distribution of DSS in HCMC. Assuming a constant extrapolation factor from DSS cases to total dengue cases and infections, this picture suggests a heterogeneous distribution of dengue risk in HCMC. A better understanding of the spatial distribution of dengue risk through population-based studies could help to inform vector control activities that are constrained by limited resources and may provide insight into some of the demographic and environmental determinants of the increasing case burden.

The rational implementation of dengue vaccines and treatment interventions can be guided by detailed clinical and epidemiological data such as described here. Although hospital-based data suffer several limitations, including a bias to more severe disease, the severe end of the disease spectrum is where the biggest health impact will be made by both vaccines and therapeutics.

## Supplementary Material

Supplementary Figure

[Supplemental figure]

## Figures and Tables

**Table 1:** Serological results for clinically diagnosed dengue patients at the Hospital for Tropical Diseases, Ho Chi Minh City, Vietnam

Year	Total			Day of testing (since onset of illness)			
				≤ 4 days		≥ 5 days	
	Dengue patients	Specimens tested	Positive (%)	Specimens tested	Positive (%)	Specimens tested	Positive (%)
2004	3,791	419	73.0	58	65.5	361	74.2
2005	2,816	655	75.4	38	65.8	617	76.0
2006	5,147	1,540	63.8	112	27.7	1,428	66.6
2007	6,754	2,769	72.3	116	35.3	2,653	74.0
2008	10,811	4,012	75.5	168	41.7	3,844	77.0
2009	10,172	2,342	68.5	150	34.7	2,192	70.8

Single plasma samples were tested for dengue virus-reactive IgM by antigen-capture ELISA.

**Table 2:** Prevalence of dengue shock syndrome among hospitalized pediatric dengue patients

Variable	*N* total	*N*	Dengue shock syndrome				
			Percent	OR	95% CI	AOR	95% CI
Total	75,057	9,392	12.51				
Sex							
Male	43,519	5128	11.78	1.0		1.0	
Female	31,538	4264	13.52	1.17*	1.12–1.22*	1.19*	1.14–1.24*
Age group							
< 1	3,318	281	8.47	0.68*	0.60–0.78*	0.62*	0.54–0.71*
1–5	16,315	1,944	11.92	1.0		1.0	
6–10	27,499	4,262	15.50	1.36*	1.28–1.44*	1.40*	1.32–1.49*
11–15	27,925	2,905	10.40	0.86*	0.81–0.91*	0.95	0.89–1.03
Hospital							
HTD	18,980	1,773	9.34	1.0		1.0	
CH1	33,542	5,515	16.44	1.91*	1.80–2.02*	1.79*	1.69–1.90*
CH2	22,535	2,104	9.34	1.0	0.94–1.07	0.96	0.89–1.03
Residence†							
HCMC	53,070	5,608	10.57	1.0		1.0	
Province	20,340	3,677	18.08	1.87*	1.79–1.95*	1.79*	1.69–1.90*
Season‡							
Dry	19,549	2,612	13.36	1.0		1.0	
Wet	55,508	6,780	12.21	0.90*	0.86–0.95*	0.96	0.91–1.0

HTD = Hospital for Tropical Disease; CH1 = Children's Hospital Number 1; CH2 = Children's Hospital Number 2; HCMC = Ho Chi Minh City; OR = odds ratio; AOR = adjusted odds ratio (adjusted for sex, age group, hospital, location of admission, year, and season of admission).

* Values are significant at *P* < 0.001.

† Location of residence was missing for 1,651 patients and imputed for multivariate analysis.

‡ Dry season is January to June, and wet season is July to December.

**Table 3:** Mortality among hospitalized pediatric dengue patients

Variable	*N* total	Mortality among all dengue cases					
		*N*	Percent	OR	95% CI	AOR	95% CI
Total	75,057	150	0.20				
Sex							
Male	43,519	70	0.16	1.0		1.0	
Female	31,538	80	0.25	1.58*	1.14–2.18*	1.57*	1.14–2.17*
Age group							
< 1	3,318	11	0.33	0.84	0.44–1.60	0.73	0.38–1.38
1–5	16,315	64	0.39	1.0		1.0	
6–10	27,499	53	0.19	0.49*	0.34–0.71*	0.52*	0.36–0.75*
11–15	27,925	22	0.08	0.20*	0.12–0.33*	0.27*	0.16–0.44*
Hospital							
HTD	18,980	13	0.07	1.0		1.0	
CH1	33,542	121	0.36	5.28*	2.98–9.36*	3.60*	2.00–6.49*
CH2	22,535	16	0.07	1.04	0.50–2.15	0.87	0.41–1.85
Residence†							
HCMC	53,070	48	0.09	1.0		1.0	
Province	20,340	102	0.50	5.57*	3.95–7.85*	3.98*	2.80–5.66*
Season‡							
Dry	19,549	50	0.26	1.0		1.0	
Wet	55,508	100	0.18	0.70§	0.50–0.99	0.88	0.62–1.24

HTD = Hospital for Tropical Disease; CH1 = Children's Hospital Number 1; CH2 = Children's Hospital Number 2; HCMC = Ho Chi Minh City; OR = odds ratio; AOR = adjusted odds ratio (adjusted for sex, age group, hospital, location of admission, year, and season of admission).

* Values are significant at *P* < 0.001.

† Location of residence was missing for 1,651 patients and imputed for multivariate analysis.

‡ Dry season is January to June, and wet season is July to December.

§ Value significant at *P* = 0.05.

**Table 4:** Mortality among pediatric patients with dengue shock syndrome

Variable	*N* DSS	Mortality among DSS cases					
		*N*	Percent	OR	95% CI	AOR	95% CI
Total	9,392	131	1.39				
Sex							
Male	5,128	56	1.09	1.0		1.0	
Female	4,264	75	1.76	1.62*	1.14–2.30*	1.51*	1.06–2.15*
Age group							
< 1	281	8	2.85	0.95	0.45–2.02	1.03	0.48–2.20
1–5	1,944	58	2.98	1.0		1.0	
6–10	4,262	46	1.08	0.35*	0.24–0.52*	0.40*	0.27–0.59*
11–15	2,905	19	0.65	0.21*	0.13–0.36*	0.28*	0.16–0.48*
Hospital							
HTD	1,773	12	0.68	1.0		1.0	
CH1	5,515	104	1.89	2.82*	1.55–5.14*	1.94*	1.04–3.61*
CH2	2,104	15	0.71	1.05	0.49–2.26	0.87	0.40–1.90
Residence†							
HCMC	5,608	43	0.77	1.0		1.0	
Province	3,677	88	2.39	3.17*	2.20–4.58*	2.10*	1.43–3.10*
Season‡							
Dry	2,612	47	1.80	1.0		1.0	
Wet	6,780	84	1.24	0.68§	0.48–0.98	0.80	0.55–1.15

HTD = Hospital for Tropical Disease; CH1 = Children's Hospital Number 1; CH2 = Children's Hospital

Number 2; HCMC = Ho Chi Minh City; OR = odds ratio; AOR = adjusted odds ratio (adjusted for sex, age group, hospital, location of admission, year, and season of admission).

* Values are significant at *P* < 0.001.

† Location of residence was missing for 1,651 patients and imputed for multivariate analysis.

‡ Dry season is January to June, and wet season is July to December.

§ Value is significant at *P* = 0.04.
