# Supplementary material for: Epidemiological Factors Associated with Dengue Shock Syndrome and Mortality in Hospitalized Dengue Patients in Ho Chi Minh City, Vietnam
Source: Am J Trop Med Hyg. 2011 Jan 5;84(1):127–34. doi: 10.4269/ajtmh.2011.10-0476 (PMC3005500; doi:10.4269/ajtmh.2011.10-0476)
Supplement: Supplementary Figure [file SD2.pdf]

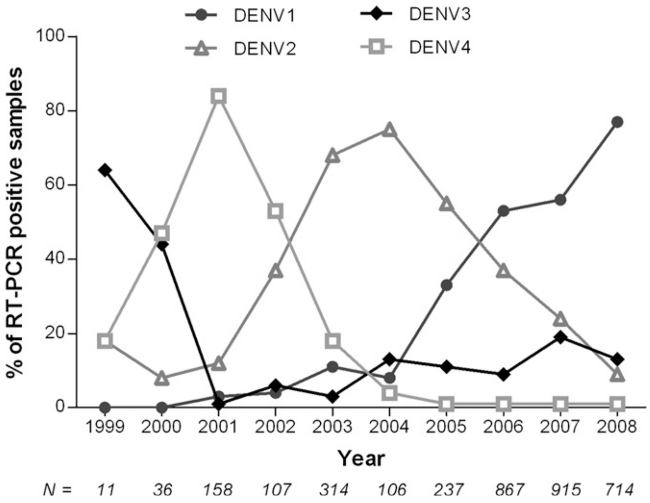

SUPPLEMENTAL FIGURE 1. Relative dengue virus serotype prevalence in patients admitted to the Hospital for Tropical Disease from 1999 to 2008. Acute blood samples from adult and pediatric dengue patients enrolled in clinical studies at HTD since 1999 were analyzed by real-time reverse transcriptase polymerase chain reaction (RT-PCR), and the infecting dengue virus (DENV) serotype was determined. Lines show the proportion of samples tested that were positive for each serotype each year. Numbers in italics under the figure show the total number of samples tested by RT-PCR each year.
