# Supplementary material for: Epidemiological Factors Associated with Dengue Shock Syndrome and Mortality in Hospitalized Dengue Patients in Ho Chi Minh City, Vietnam
Source: Am J Trop Med Hyg. 2011 Jan 5;84(1):127–34. doi: 10.4269/ajtmh.2011.10-0476 (PMC3005500; doi:10.4269/ajtmh.2011.10-0476)
Supplement: [Supplemental figure] [file supp_84_1_127__index.html]

 Epidemiological Factors Associated with Dengue Shock Syndrome and Mortality in Hospitalized Dengue Patients in Ho Chi Minh City, Vietnam -- Anders et al. 84 (1): 127 Data Supplement - Supplemental figure -- American Journal of Tropical Medicine and Hygiene **Epidemiological Factors Associated with Dengue Shock Syndrome and Mortality in Hospitalized Dengue Patients in Ho Chi Minh City, Vietnam**  
 Am J Trop Med Hyg Anders et al. 84: 127

## Supplemental figure

**Files in this Data Supplement:**

- Supplemental figure
